# Prevalence and antibiotic resistance profiles of cerebrospinal fluid pathogens in children with acute bacterial meningitis in Yunnan province, China, 2012-2015

**DOI:** 10.1371/journal.pone.0180161

**Published:** 2017-06-29

**Authors:** Hongchao Jiang, Min Su, Liyue Kui, Hailin Huang, Lijuan Qiu, Li Li, Jing Ma, Tingyi Du, Mao Fan, Qiangming Sun, Xiaomei Liu

**Affiliations:** 1 The Affiliated Children’s Hospital of Kunming Medical University, Kunming, P.R. China; 2 Institute of Medical Biology, Chinese Academy of Medical Sciences and Peking Union Medical College, Kunming, P.R. China; 3 Yunnan Key Laboratory of Vaccine Research & Development on Severe Infectious Diseases, Kunming, PR China; Emory University School of Medicine, UNITED STATES

## Abstract

Acute bacterial meningitis is still considered one of the most dangerous infectious diseases in children. To investigate the prevalence and antibiotic resistance profiles of cerebrospinal fluid (CSF) pathogens in children with acute bacterial meningitis in Southwest China, CSF samples from 179 meningitis patients (3 days to 12 years old) with positive culture results were collected from 2012 to 2015. Isolated pathogens were identified using the Vitek-32 system. Gram stain results were used to guide subcultures and susceptibility testing. The antimicrobial susceptibility of isolates was determined using the disc diffusion method. Of the isolates, 50.8% were Gram-positive bacteria, and 49.2% were Gram-negative bacteria. The most prevalent pathogens were *E*. *coli* (28.5%), *Streptococcus pneumoniae* (17.8%), *Staphylococcus epidermidis* (10.0%), *Haemophilus influenzae type b* (9.5%), and *group B streptococcus* (7.2%). In young infants aged ≤3 months, *E*. *coli* was the organism most frequently isolated from CSF (39/76; 51.3%), followed by *group B streptococcus* (13/76; 17.1%) and *Streptococcus pneumoniae* (8/76; 10.5%). However, in young infants aged >3 months, the most frequently isolated organism was *Streptococcus pneumoniae* (24/103; 23.3%), followed by *Staphylococcus epidermidis* (18/103; 17.5%) and *Haemophilus influenzae type b* (16/103; 15.5%). Antimicrobial susceptibility tests indicated that for *E*. *coli* isolates, the susceptibility rates to aminoglycosides ranged from 56.8% to 100.0%, among them, amikacin was identified as the most effective against *E*. *coli*. As for cephalosporins, the susceptibility rates ranged from 29.4% to 78.4%, and cefoxitin was identified as the most effective cephalosporin. In addition, the susceptibility rates of piperacillin/tazobactam and imipenem against E. coli were 86.3% and 100%. Meanwhile, the susceptibility rates of *Streptococcus pneumoniae* isolates to penicillin G, erythromycin, chloramphenicol, ceftriaxone and tetracycline were 68.8%, 0.0%, 87.5%, 81.3% and 0.0%, respectively. Gentamycin, ofloxacin, linezolid and vancomycin were identified as the most effective antibiotics for *Streptococcus pneumoniae*, each with susceptibility rates of 100%. It was notable that other emerging pathogens, such as *Listeria monocytogenes* and *group D streptococcus*, cannot be underestimated in meningitis.

## Introduction

Meningitis is an inflammation of the membranes (meninges) surrounding the brain and spinal cord. Most cases of bacterial meningitis occur during childhood, and acute bacterial meningitis has been found to be a fatal and urgent condition associated with a high rate of mortality and serious potential morbidity [1, 2]. Therefore, early diagnosis and appropriate antibiotic treatment are necessary to avoid further complications. It has been reported that *Streptococcus pneumoniae*, *Neisseria meningitidis*, and *Haemophilus influenzae* type b were among the most prevalent pathogens causing this disease [3–6]. However, the prevalence and etiologies of meningitis pathogens may vary during different times, in geographical regions, and according to the age of the patients [3, 7–13]. Moreover, the effectiveness of treatment may be limited due to antibiotic-resistant bacterial strains. Periodic reviews of meningitis cases are necessary.

Thus far, the epidemiology of the pathogens causing meningitis in Yunnan province has not been well characterized. Meanwhile, little has been reported regarding the antibiotic resistance patterns of prevalent bacteria in this area. This study was the first comprehensive study to investigate the etiological profile and antimicrobial resistance patterns of bacterial isolates causing meningitis in children in Yunnan province, China from January 1, 2012 to December 31, 2015. This study may help facilitate decision making regarding the vaccination of children against causative bacteria and improve evidence-based therapeutic strategies in Yunnan province, China.

## Materials and methods

### Ethical statement

All participants were informed of the study aims, and written informed consent was obtained from a parent of each patient before sample collection. This study was conducted with the approval of the institutional ethics committee of the Children’s Hospital of Kunming Medical University and was in accordance with the Declaration of Helsinki for Human Research of 1974 (last modified in 2000).

### Study area and population

The study was carried out at the Children’s Hospital of Kunming Medical University (CHKMU), the largest and only Children’s Hospital in Yunnan province, China. CHKMU is an approximately a one thousand beds tertiary medical facility located in Kunming, the capital of Yunnan Province. As an important bridge in the Greater Mekong Sub-regional Economic Cooperation, the border between the Yunnan Province and Myanmar, Laos and Vietnam extends 4061 km. In addition to providing care for patients located in the southwestern region of China, this teaching hospital also attends to referral cases from the countries of southern Asia (Vietnam, Laos and Thailand). The hospital attends to an average of 1,500,000 outpatient cases and 63,000 inpatient cases each year.

### Study subjects and data collection

The study was conducted from January 2012 to December 2015. Confirmed cases of meningitis in the Children’s Hospital of Kunming Medical University were identified according to the case definition for acute bacterial meningitis. Overall, 179 confirmed acute meningitis patients whose parent or legal guardian provided written informed consent were enrolled in this study. Patient information, including age, sex, and clinical symptoms, was recorded at the time of admission. All patients’ clinical diagnoses and carriage and antibiotic susceptibility profile of isolated organisms were collected and analyzed according to the guidelines of the hospital ethical committee.

### Clinical specimen collection

Only one representative CSF sample from each patient was included, and consecutive CSF samples from the same patient were ignored for the purposes of this study. The puncture site was disinfected with 70% alcohol and a 2% tincture of iodine before collecting approximately 3 ml of CSF from patients. Lumbar puncture was performed aseptically on the patients, and cerebrospinal fluid (CSF) samples were collected in sterile screw-capped containers.

### Case definitions

A total of 179 confirmed case of acute bacterial meningitis met the following definition and were included in this study: patients with a clinical meningeal syndrome and a positive bacterial agent isolated by CSF culture [14, 15].

### Organism identification and speciation

Immediately after collection, each CSF specimen was centrifuged at 1500 rpm for 15 minutes. The supernatant was removed, and the sediment was analyzed by Gram a stains if the patient’s white blood cell count was >10/mm^3^. In addition, cerebrospinal fluid (CSF) specimens were inoculated into PEDS Plus bottles and cultivated using the BD BACTEC^™^ FX system (BD Diagnostics, Sparks, MD). Gram stain results were used to guide subcultures and susceptibility testing, which was performed using the disc diffusion method. Specimens were subcultured onto 5% sheep blood agar and chocolate agar plates. All culture plates were incubated at 37°C for 24–48 hours in a 5% carbon dioxide environment and at room temperature for bacterial organisms, respectively. Bacterial isolates were identified using the Vitek-32 system (BioMerieux).

### Antibiotic susceptibility tests

Antibiotic susceptibilities were determined using the Kirby-Bauer disc diffusion method on Mueller-Hinton agar, interpreted according to the Clinical and Laboratory Standards Institute (CLSI) guidelines and quality-controlled using *Escherichia coli* ATCC25922 and *Staphylococcus aureus* ATCC25923. The antibiotics tested were obtained from the Oxoid Company, located in the UK. Isolates showing intermediate levels of susceptibility were classified as nonsusceptible.

### Statistical analysis

WHONET, a software developed by the World Health Organization Collaborating Centre for Surveillance of Antimicrobial Resistance, was used to extract data from the CSF testing results. The extracted data were cleaned by visual inspection and preliminary frequency analysis of the raw data set and analyzed using SPSS software version 13.0. Chi-square tests were used to calculate rates and percentages. The significance level was defined as *p <* 0.05.

## Results

### The distribution of confirmed meningitis cases from January 2012 to December 2015

**Fig 1:**
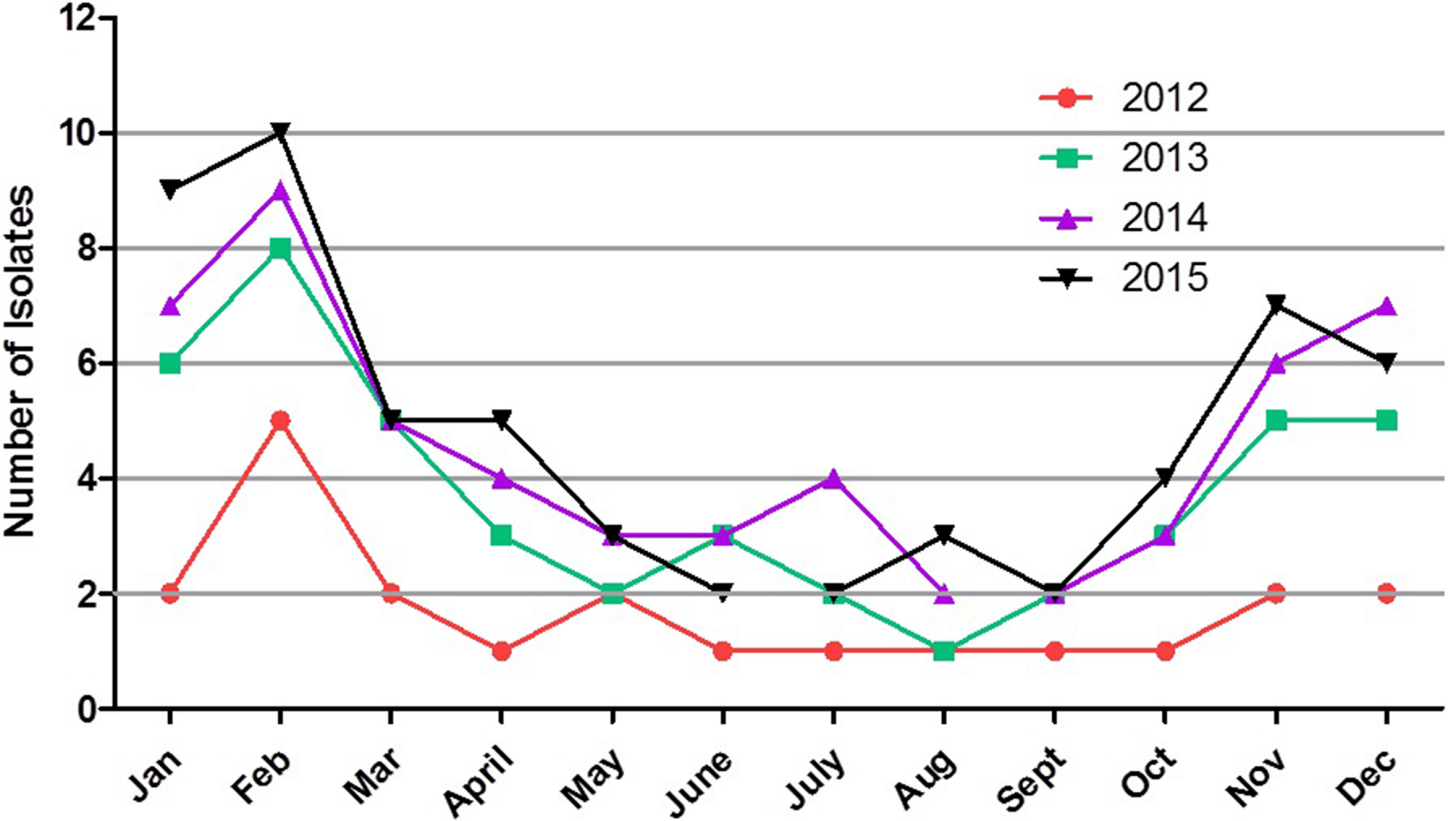
Seasonal variation in the number of confirmed meningitis cases was examined from 2012 to 2015. A greater number of confirmed meningitis cases occurred during 2015 than occurred during any other year. The highest number of confirmed meningitis cases occurred during February 2015. The monthly prevalence of confirmed meningitis was, for the most part, lower in 2012, except for during the month of February. It was notable that the seasonal distribution indicated that the number of positive CSF cultures decreased during the rainy season (May—October) in Southwest China.

The numbers of annual confirmed meningitis cases were 21 in 2012, 45 in 2013, 55 in 2014 and 58 in 2015 in Children’s Hospital of Kunming Medical University (Table 1). There was a steady growth in the number of annual meningitis cases over the four-year period. The prevalence of the pathogens isolated from the patients’ CSF by year is listed in Table 1. *Streptococcus pneumoniae* was the most frequently isolated Gram-positive strain, but the percentage of the total isolates accounted for by *Streptococcus pneumoniae* each year varied during 2012–2015. Among the Gram-negative stains, *E*. *coli* was most frequently isolated, and the percentage of the total isolates of *E*. *coli* increased stably during 2012–2015. Seasonal variation in the number of confirmed meningitis cases was examined over the four-year period (Fig 1). A greater number of confirmed meningitis cases occurred during 2015 than occurred during any other year. The highest number of confirmed meningitis cases occurred during February 2015. The monthly prevalence of confirmed meningitis was, for the most part, lower in 2012, except for during the month of February. It was notable that the seasonal distribution indicated that the number of positive CSF cultures decreased during the rainy season (May—October) in Yunnan province, China.

**Table 1:** Yearly distribution of meningitis pathogens.

Pathogens	2012 N (%)	2013 N (%)	2014 N (%)	2015 N (%)	Total N (%)
Gram-positive organisms					91(50.8)
*Streptococcus pneumoniae*	4(2.2)	10(5.6)	8(4.4)	10(5.6)	32(17.8)
*Staphylococcus epidermidis*	2(1.1)	5(2.9)	6(3.3)	5(2.9)	18(10.0)
*Group B Streptococcus*	2(1.1)	2(1.1)	4(2.2)	5(2.9)	13(7.2)
*Staphylococcus haemolyticus*	2(1.1)	2(1.1)	3(1.6)	1(0.6)	8(4.4)
*Group D Streptococcus*	1(0.6)	1(0.6)	2(1.1)	2(1.1)	6(3.4)
*Staphylococcus aureus*	1(0.6)	1(0.6)	2(1.1)	1(0.6)	5(2.9)
*Staphylococcus hominis*	0(0.0)	2(1.1)	1(0.6)	2(1.1)	5(2.9)
*Listeria monocytogenes*	0(0.0)	0(0.0)	2(1.1)	2(1.1)	4(2.2)
Gram-negative organisms					88(49.2)
*E*. *coli*	5(2.9)	13(7.3)	15(8.4)	18 (10.1)	51(28.5)
*Haemophilusinfluenzaetype b*	2(1.1)	5(2.9)	4(2.2)	6(3.3)	17(9.5)
*S*. *entericaserovarTyphimurium*	1(0.6)	2(1.1)	2(1.1)	2(1.1)	7(3.9)
*Klebsiellapneumoniae*	0(0.0)	1(0.6)	1(0.6)	1(0.6)	3(1.7)
*Pseudomonas aeruginosa*	1(0.6)	1(0.6)	1(0.6)	0(0.0)	3(1.7)
*Moraxella catarrhalis*	0(0.0)	0(0.0)	2(1.1)	1(0.6)	3(1.7)
*Acinetobacterbaumannii*	0(0.0)	0(0.0)	1(0.6)	1(0.6)	2(1.1)
*Acinetobacterlwoffii*	0(0.0)	0(0.0)	1(0.6)	1(0.6)	2(1.1)
Total	21(11.7)	45(25.1)	55(30.7)	58(32.5)	179(100.0)

### Prevalence of CSF pathogens in pediatric acute meningitis patients in Yunnan province, China

During the 4 years of observation, 179 confirmed acute bacterial meningitis cases were reported based on positive cerebrospinal fluid culture results. These meningitis patients included 120 males (67.1%) and 59 females (32.9%) ranging from 3 days to 12 years old (mean age = 1.8 years). Clinical manifestations observed during hospitalization are presented in Table 2. Fever, vomiting, and meningeal irritation were the most prevalent symptoms in patients. All patients (100%) had fever. Across all age groups, 91/179 (50.8%) of isolates were Gram-positive bacteria, 88/179 (49.2%) were Gram-negative bacteria. The main prevalent organisms isolated from CSF cultures were *E*. *coli* (28.5%), *Streptococcus pneumoniae* (17.8%), *Staphylococcus epidermidis* (10.0%), *Haemophilus influenzae* type b (9.5%), and *group B streptococcus* (7.2%). The prevalence of other pathogens isolated from the patients’ CSF samples is listed in Table 3. In young infants aged ≤3 months, *E*. *coli* was the organism most frequently isolated from CSF (39/76; 51.3%), followed by *group B streptococcus* (13/76; 17.1%) and *Streptococcus pneumoniae* (8/76; 10.5%). However, in young infants aged >3 months, the most frequently isolated organism was *Streptococcus pneumoniae* (24/103; 23.3%), followed by *Staphylococcus epidermidis* (18/103; 17.5%) and *Haemophilus influenzae* type b (16/103; 15.5%) (Table 3).

**Table 2:** Clinical manifestations of pediatric patients with acute meningitis from 2012–2015.

Clinical Symptoms	≤28 days	>28days ≤3 months	>3months ≤1 ages	>1ages ≤3ages	>3ages ≤12 ages	Number of Patients	Occurrence (%)
Fever	40	36	53	27	23	179	100
Vomiting	32	31	43	14	15	135	75.4
Meninges irritation	28	27	39	11	11	116	64.8
Seizure	18	17	23	9	8	75	41.8
Lethargy	16	15	21	7	6	65	36.3
Headache	3	3	6	8	7	27	15.1
Stupor	5	3	5	3	2	18	10.1
Coma	3	2	3	1	1	10	5.6

**Table 3:** Frequency of bacteria isolated from patients’ CSF of different age.

Isolate	≤28 days a/b (%)	>28days —3 months a/b (%)	>3months ≤1 ages a/b (%)	>1ages ≤3ages a/b (%)	>3ages ≤12 ages a/b (%)	All ages a/b (%)
Gram-positive organisms						91/179 (50.8)
*Streptococcus pneumoniae*	4/179(2.2)	4/179 (2.2)	16/179 (8.8)	5/179 (1.1)	3/179 (0.0)	32/179 (17.8)
*Staphylococcus epidermidis*	0/179(0.0)	0/179 (0.0)	8/179 (4.4)	3/179 (1.7)	7/179 (3.9)	18/179 (10.0)
Group B *Streptococcus*	6/179 (3.3)	7/179 (3.9)	0/179 (0.0)	0/179 (0.0)	0/179 (0.0)	13/179 (7.2)
*Staphylococcus haemolyticus*	5/179 (2.9)	0/179 (0.0)	1/179 (0.6)	1/179 (0.6)	1/179 (0.6)	8/179 (4.4)
Group D *Streptococcus*	0/179 (0.0)	0/179 (0.0)	2/179 (1.1)	2/179 (1.1)	2/179 (1.1)	6/179 (3.4)
*Staphylococcus aureus*	0/179 (0.0)	1/179 (0.6)	1/179 (0.6)	1/179 (0.6)	2/179 (1.1)	5/179 (2.9)
*Staphylococcus hominis*	0/179 (0.0)	2/179 (1.1)	1/179 (0.6)	1/179 (0.6)	1/179 (0.6)	5/179 (2.9)
*Listeria monocytogenes*	0/179 (0.0)	0/179 (0.0)	2/179 (1.1)	2/179 (1.1)	0/179 (0.0)	4/179 (2.2)
Gram-negative organisms						88/179 (49.2)
*E*. *coli*	20/179 (11.3)	19/179 (8.8)	11/179 (4.4)	0/179 (0.0)	1/179 (0.6)	51/179 (28.5)
*Haemophilus influenzae* type b	1/179 (0.6)	0/179 (0.0)	8/179 (4.4)	8/179 (4.4)	0/179 (0.0)	17/179 (9.5)
*S*.*entericaserovar Typhimurium*	1/179 (0.6)	0/179 (0.0)	2/179 (1.1)	2/179 (1.1)	2/179 (1.1)	7/179 (3.9)
*Klebsiella pneumoniae*	1/179 (0.6)	2/179 (1.1)	0/179 (0.0)	0/179 (0.0)	0/179 (0.0)	3/179 (1.7)
*Pseudomonas aeruginosa*	2/179 (1.1)	1/179 (0.6)	0/179 (0.0)	0/179 (0.0)	0/179 (0.0)	3/179 (1.7)
*Moraxella catarrhalis*	0/179 (0.0)	0/179 (0.0)	0/179 (0.0)	0/179 (0.0)	3/179 (1.7)	3/179 (1.7)
*Acinetobacter baumannii*	0/179 (0.0)	0/179 (0.0)	1/179 (0.6)	1/179 (0.6)	0/179 (0.0)	2/179 (1.1)
*Acinetobacter lwoffii*	0/179 (0.0)	0/179 (0.0)	0/179 (0.0)	1/179 (0.6)	1/179 (0.6)	2/179 (1.1)
Total	40/179 (22.4)	36/179 (20.1)	53/179 (29.6)	27/179 (15.1)	23/179 (12.8)	179/179 (100.0)

a/b (%), number of kind of bacteria isolated from patients’ CSF /total number of bacteria isolated from patients’ CSF (percentage of kind of bacteria).

### Antibiotic resistance patterns of Gram-positive pathogens isolated from CSF

The in vitro Gram-positive antimicrobial activities of the tested antibiotics are summarized in Table 4. The susceptibility rates of *Streptococcus pneumoniae* isolates to penicillin G, erythromycin, chloramphenicol, ceftriaxone and tetracycline were 68.8%, 0.0%, 87.5%, 81.3% and 0.0%, respectively. Gentamycin, ofloxacin, linezolid and vancomycin were identified as the most effective antibiotics for *Streptococcus pneumoniae*, each with susceptibility rates of 100% (Table 4). For *Staphylococcus epidermidis* isolates, the susceptibility rates to penicillin G, erythromycin, chloramphenicol and tetracycline were 0.0%, 5.6%, 83.3% and 33.3%, respectively. Vancomycin, linezolid and rifampicin were identified as the most effective antibiotics for *Staphylococcus epidermidis*, with susceptibility rates of 100%, 100% and 88.9%, respectively (Table 4). For group B streptococcus isolates, the susceptibility rates to penicillin G, erythromycin, chloramphenicol, ceftriaxone and tetracycline were 100.0%, 7.7%, 84.6%, 100% and 0.0%, respectively. Vancomycin, ceftriaxone, meropenem, ampicillin, linezolid and Penicillin G were identified as the most effective antibiotics for group B streptococcus, each with susceptibility rates of 100% (Table 4).

**Table 4:** Antimicrobial susceptibility of gram positive bacterial pathogens isolated from CSF culture.

Antimicrobial	*Streptococcus pneumoniae* *a/b (%)*	*Staphylococcus epidermidis* *a/b (%)*	*GroupB* *Streptococcus* *a/b (%)*	*Staphylococcus haemolyticus* *a/b (%)*	*GroupD Streptococcus* *a/b (%)*	*Staphylococcus Aureus* *a/b (%)*	*Staphylococcus Hominis* *a/b (%)*	*Listeria monocytogenes* *a/b (%)*
Penicillin G	20/32(68.8)	0/18(0.0)	13/13(100.0)	0/8(0.0)	0/6(0.0)	0/5(0.0)	1/5 (20.0)	0/4(0.0)
Oxacillin	–	6/18(33.3)	–	0/8(0.0)	0/6 (0.0)	3/5 (60.0)	2/5(40.0)	3/4 (75.0)
Gentamicin	32/32 (100.0)	14/18(77.8)	–	4/8(50.0)	0/6 (0.0)	5/5 (100.0)	5/5(100.0)	3/4 (75.0)
Rifampicin	–	16/18(88.9)	–	6/8(75.0)	4/6 (66.7)	5/5 (100.0)	5/5 (100.0)	–
Ciprofloxacin	–	10/18(55.6)	9/13(69.2)	2/8(25.0)	4/6 (66.7)	5/5 (100.0)	3/5 (60.0)	4/4 (100.0)
Moxifloxacin	–	8/18(44.4)	–	2/8(25.0)	–	5/5 (100.0)	3/5(60.0)	–
Norfloxacin	–	10/18(55.6)	–	1/8(12.5)	4/6 (66.7)	5/5 (100.0)	3/5(60.0)	4/4 (100.0)
Ofloxacin	32/32(100.0)	8/18(44.4)	11/13(84.6)	0/8(0.0)	3/6 (50.0)	5/5 (100.0)	3/5(60.0)	4/4 (100.0)
Levofloxacin	28/32(87.5)	10/18(55.6)	10/13(76.9)	1/8(12.5)	3/6 (50.0)	5/5 (100.0)	3/5(60.0	4/4 (100.0)
Co-trimoxazole	0/32(0.0)	9/18(50.0)	–	4/8(50.0)	4/6 (66.7)	3/5 (60.0)	2/5(40.0)	–
Clindamycin	0/32(0.0)	11/18(61.1)	3/13(23.1)	3/8(37.5)	4/6 (66.7)	1/5 (20.0)	2/5(40.0)	–
Erythromycin	0/32(0.0)	1/18(5.6)	1/13(7.7)	0/8(0.0)	0/6 (0.0)	1/5 (20.0)	0/5(0.0)	2/4 (50.0)
Linezolid	32/32(100.0)	18/18(100.0)	13/13(100.0)	8/8(100.0)	6/6 (100.0)	5/5 (100.0)	5/5(100.0)	4/4 (100.0)
Vancomycin	32/32(100.0)	18/18(100.0)	13/13(100.0)	8/8(100.0)	5/6 (83.3)	5/5 (100.0)	5/5 (100.0)	4/4 (100.0)
Chloramphenicol	28/32(87.5)	15/18(83.3)	11/13(84.6)	7/8(87.5)	4/6 (66.7)	3/5 (60.0)	4/5 (80.0)	–
Tetracycline	0/32(0.0)	6/18(33.3)	0/13(0.0)	4/8(50.0)	1/6 (16.7)	1/5 (20.0)	3/5 (60.0)	–
Ampicillin	–	0/18(0.0)	13/13(100.0)	0/8(0.0)	3/6 (50.0)	0/5 (0.0)	1/5 (20.0)	2/4 (50.0)
Amoxicillin+clavulanate	–	5/18(27.7)	–	2/8(25.0)	–	2/5 (40.0)	2/5 (40.0)	–
Ceftriaxone	26/32(81.3)	–	13/13(100.0)	–	0/6 (0.0)	–	–	4/4 (100.0)
Meropenem	24/32(75.0)	–	13/13(100.0)	–	–	–	–	–
Azithromycin	–	0/18(0.0)	–	0/8(0.0)	–	0/5 (0.0)	0/5 (0.0)	–
Clarithromycin	–	3/18(16.6)	–	2/8(25.0)	–	4/5 (80.0)	3/5 (60.0)	–
Total	32	18	13	8	6	5	5	4

a/b (%), number susceptible/number tested (percentage susceptible).

Antibiotics were not tested against all organisms with dash (–).

### Antimicrobial susceptibility of Gram-negative pathogens isolated from CSF

The in vitro Gram-negative antimicrobial activity of the tested antibiotics are summarized in Table 5. The results showed that more than 95% of Gram-negative bacteria were resistant to ampicillin. For *E*. *coli* isolates, the susceptibility rates to aminoglycosides ranged from 56.8% to 100.0%, whereas the susceptibility rates to cephalosporins ranged from 29.4% to 78.4%. Among the tested aminoglycosides, amikacin was identified as the most effective against *E*. *coli*, with a susceptibility rate of 100.0%, and the most effective cephalosporin for the treatment of *E*. *coli* was identified as cefoxitin, with a susceptibility rate of 78.4%. In this study, piperacillin/tazobactam and imipenem were identified as the most effective antibiotics against *E*. *coli*, with susceptibility rates of 86.3% and 100%, respectively. For *Haemophilus influenzae* type b, susceptibility rates to aminoglycosides ranged from 23.5% to 88.2%, whereas susceptibility rates to cephalosporins ranged from 35.2% to 94.1%. Among the tested aminoglycosides, gentamicin was identified as the most effective against *Haemophilus influenzae* type b, with a susceptibility rate of 88.2%, and the most effective cephalosporin for the treatment of *Haemophilus influenzae* type b was identified as cefepime, with a susceptibility rate of 94.1%. In this study, ciprofloxacin, cefepime and imipenem were identified as the most effective antibiotics against *Haemophilus influenzae* type b, with susceptibility rates of 94.1%, 94.1% and 100%, respectively (Table 5).

**Table 5:** Antimicrobial susceptibility of gram negative bacterial pathogens isolated from CSF culture.

Antimicrobial	*E*. *coli* *a/b (%)*	*Haemophilus influenzae type b* *a/b (%)*	*S*. *enterica serovar Typhimurium* *a/b (%)*	*Klebsiella pneumoniae* *a/b (%)*	*Pseudomonas aeruginosa* *a/b (%)*	*Moraxella catarrhalis* *a/b (%)*	*Acinetobacter baumannii* *a/b (%)*	*Acinetobacter lwoffii* *a/b (%)*
Ampicillin	2/51(3.9)	1/17(5.8)	0/7(0.0)	0/3(0.0)	–	0/3(0.0)	–	–
Piperacillin	8/51(15.7)	–	–	–	2/3(66.7)	–	1/2(50.0)	1/2(50.0)
Ampicillin/sulbactam	15/51(29.4)	–	–	3/3(100.0)	1/3(33.3)	2/3(66.6)	–	–
Piperacillin/Tazobactam	44/51(86.3)	–	–	3/3(100.0)	3/3(100.0)	–	2/2(100.0)	1/2(50.0)
Cefoperazone	15/51(29.4)	6/17(35.2)	3/7 (42.8)	1/3(33.3)	–	2/3(66.6)	0/2(0.0)	0/2(0.0)
Cefuroxime	22/51(43.1)	14/17(82.4)	2/7(28.5)	1/3(33.3)	–	1/3(33.3)	0/2(0.0)	0/2(0.0)
Ceftazidime	34/51(66.6)	14/17(82.4)	3/7 (42.8)	1/3(33.3)	2/3(66.7)	2/3(66.6)	0/2(0.0)	0/2(0.0)
Ceftriaxone	22/51(43.1)	14/17(82.4)	2/7 (28.5)	1/3(33.3)	0/3(0.0)	1/3(33.3)	0/2(0.0)	0/2(0.0)
Cefepime	34/51(66.6)	16/17(94.1)	4/7 (57.1)	2/3(66.6)	2/3(66.7)	3/3(100.0)	0/2(0.0)	0/2(0.0)
Cefoxitin	44/51(78.4)	–	–	1/3(33.3)	–	–	–	–
Aztreonam	25/51(49.0)	–	–	–	2/3(66.7)	–	–	–
Imipenem	51/51(100.0)	17/17(100.0)	7/7(100.0)	3/3(100.0)	3/3(100.0)	3/3(100.0)	2/2(100.0)	2/2(100.0)
Meropenem	51/51(100.0)	17/17(100.0)	7/7(100.0)	3/3(100.0)	3/3(100.0)	3/3(100.0)	2/2(100.0)	2/2(100.0)
Amikacin	51/51(100.0)	4/17(23.5)	7/7(100.0)	1/3(33.3)	3/3(100.0)	3/3(100.0)	1/2(50.0)	1/2(50.0)
Gentamicin	30/51(58.8)	15/17(88.2)	6/7(85.7)	1/3(33.3)	3/3(100.0)	–	1/2(50.0)	1/2(50.0)
Tobramycin	38/51(74.5)	–	6/7(85.7)	–	3/3(100.0)	–	0/2(0.0)	0/2(0.0)
Ciprofloxacin	32/51(62.7)	16/17(94.1)	–	2/3(66.6)	3/3(100.0)	3/3(100.0)	1/2(50.0)	1/2(50.0)
Levofloxacin	30/51(58.8)	–	5/7(71.4)	2/3(66.6)	3/3(100.0)	3/3(100.0)	1/2(50.0)	1/2(50.0)
Norfloxacin	29/51(56.8)	–	6/7(85.7)	–	–	3/3(100.0)	–	–
Co-trimoxazole	16/51(31.4)	4/17(23.5)	1/7(14.2)	1/3(33.3)	–	–	0/2(0.0)	0/2(0.0)
Chloramphenicol	45/51(88.2)	17/17(100.0)	1/7(14.2)	3/3(100.0)	1/3(33.3)	–	1/2(50.0)	0/2(0.0)
Minocycline	38/51(74.5)	–	–	–	–	3/3(100.0)	–	–
Total	51	17	7	3	3	3	2	2

a/b (%), number susceptible/number tested (percentage susceptible).

Antibiotics were not tested against all organisms with dash (–).

*Acinetobacter spp*. accounted for 2.2% of all isolates. Among *Acinetobacter spp*. isolates, the susceptibility rates to aminoglycosides were less than 50.0%. In addition, all *Acinetobacter spp*. isolates were sensitive to imipenem and meropenem and resistant to ceftriaxone, ceftazidime and ceftizoxime (Table 5).

## Discussion

Acute bacterial meningitis remains a major health problem in children and newborn infants worldwide and, therefore, requires early diagnosis and aggressive therapy [2, 5, 16]. Despite the availability of potent newer antibiotics, the mortality rate associated with acute bacterial meningitis remains very high in some developing countries, ranging from 16–32% [16–20]. Meanwhile, according to the results of extensive studies of the etiology of meningitis that have been conducted in Nepal, French Guiana, the North American Arctic and northern Togo, *Streptococcus pneumoniae* and *Haemophilus influenzae* type b were the pathogens most frequently isolated from the CSF of pediatric bacterial meningitis cases [6, 17, 21–23]. Nevertheless, a report from Turkey showed that *Neisseria meningitides* serogroup W135 was the dominant organism isolated from children with bacterial meningitis [24]. Thus, the etiological pathogens responsible for meningitis have been found to be relatively diverse. In this study, the organisms most frequently isolated from the CSF of meningitis patients in Yunnan province, China during 2012 to 2015 were *E*. *coli* (28.5%), *Streptococcus pneumoniae* (17.8%), *Staphylococcus epidermidis* (10.0%), *Haemophilus influenzae* type b (9.5%), and group B streptococcus (7.2%).

In recent years, two main changes have been observed in the epidemiology of acute bacterial meningitis in children [25]. The first change was that the prevalence of *Streptococcus pneumoniae*, *Haemophilus influenzae* type b and *Neisseria meningitides* significantly decreased in some developed countries due to the administration of vaccines [26, 27]. Similarly, the incidence of acute bacterial meningitis caused by with *Streptococcus pneumoniae*, *Haemophilus influenzae* type b and *Neisseria meningitides* also decreased in Yunnan province, China, where vaccinations were partially performed against these bacteria. In this study, *Neisseria meningitidis* was identified in 0% of the tested isolates. This finding is in accordance with studies performed in developed countries where the meningitis vaccine has been administered [21]. The second change in the epidemiology of acute bacterial meningitis was the increase in resistant strains of pneumococcus worldwide. One factor that may have contributed to the increasing prevalence of antibiotic resistant meningitis was the use of antibiotics before hospital admission, a common practice in many developing countries [28, 29].

Our study identified *E*. *coli* as most prevalent pathogen, which was identified in 28.5% of isolates obtained from the children included in this study (Table 3); similar results have been reported in developing countries [30]. Another prevalent bacteria was *Streptococcus pneumoniae* (17.8%), which was likely caused by endogenous transmission of *Streptococcus pneumoniae* from the nasopharynx to the meninges, especially during the dry season when cracks and injuries tend to occur in the nasopharynx [15, 31]. The seasonal distribution of confirmed meningitis cases indicated that an increase in the number of cases occurred from December to January, with the peak number of cases in 2012 identified in February. The highest number of cases was identified in February of 2015, followed by February of 2013. This trend was not surprising, as the dry season reaches its peak during these months, thus providing optimal conditions for the disruption of mucosal defenses and resulting in increased susceptibility to meningitis.

*Listeria monocytogenes* and *group D streptococcus* have been previously identified as important pathogens in the etiology of bacterial meningitis in other areas. These bacteria were also isolated from the CSF samples cultured in our study. The role of the aforementioned pathogens cannot be underestimated in the future treatment and management of meningitis.

In addition, our research showed that *E*. *coli* (39/76; 51.3%) was the most frequently isolated pathogen in young infants aged ≤3 months, while *Streptococcus pneumoniae* (24/103; 23.3%) was the most frequently isolated pathogen in young infants aged >3 months. Our result were quite different than those reported by previous studies, which identified *group B streptococcus* as the pathogen most frequently isolated from the CSF of pediatric patients ≤ 30 days of age (60/202; 29.7%), followed by *Streptococcus pneumoniae* (47/202; 23.3%). Therefore, *Group B Streptococcus* is became one of important emerging cause of neonatal meningitisin in sub-Saharan Africa [32].

In this study, we investigated culture-positive bacterial meningitis in children with bacterial meningitis symptoms, and those with negative culture results were excluded. As shown in Tables 4 and 5, all *E*. *coli* isolates exhibited intermediate resistance against third generation cephalosporins which show similar datas to previous reports. These results represent an alarming and disturbing rate of resistance in *E*. *coli* species in Yunnan province, China. Emerging resistance to third generation cephalosporins was also identified as critically concerning in recent studies because increased use of cephalosporins is likely to result in a rapid increase in the prevalence of extended spectrum-lactamases (ESBLs)-producing strains. In our study, similar to the results of a previous report, antibiotic resistance among *E*. *coli* isolates was frequently identified. High resistance rates to ceftriaxone and ceftizoxime among these organisms were suggestive of the presence of extended spectrum-lactamases (ESBLs)-producing strains. Therefore, the use of piperacillin/tazobactam for the treatment of meningitis caused by *E*. *coli* may be helpful. Moreover, antimicrobial susceptibility testing of the isolates demonstrated that all of the tested *Streptococcus pneumoniae* isolates were 100% susceptible to erythromycin, gentamycin, ofloxacin, linezolid and vancomycin. Of the *Streptococcus pneumoniae* isolates, 87.5% were susceptible to chloramphenicol, whereas 68.8% were susceptible to penicillin. Previous studies have, however, documented pneumococcal penicillin resistance rates ranging from 8%-31% and chloramphenicol resistance rates of 5–20.6% [6, 33–35]. In addition, gentamicin may not be chosen as a treatment option against *Streptococcus pneumonia* infection in children. It is not only because its narrow therapeutic index and inability to measure therapeutic drug levels, but also the risks of ototoxicity and nephrotoxicity. Therefore, ceftriaxone has been considered as the first choice treatment for *Streptococcus pneumoniae* meningitis, with penicillin considered as an alternative. In addition, the results of this study indicated that *Haemophilus influenzae* type b isolates exhibited considerable resistance to third generation cephalosporins but susceptibility to cefepime and imipenem. Regarding group B streptococcus, no resistance was identified against vancomycin, ampicillin, ceftriaxone and penicillin. Our study revealed that the emergence of resistant strains should be continuously monitored, and then provide reference for the selection of appropriate antibiotics.

## Supporting information

S1 Table Yearly distribution of meningitis pathogens. (DOC)

S2 Table Clinical manifestations of pediatric patients with acute meningitis from 2012–2015. (DOC)

S3 Table Frequency of bacteria isolated from patients’ CSF of different age. (DOC)

S4 Table Antimicrobial susceptibility of gram positive bacterial pathogens isolated from CSF culture. (DOC)

S5 Table Antimicrobial susceptibility of gram negative bacterial pathogens isolated from CSF culture. (DOC)
