# Supplementary material for: Prevalence and antibiotic resistance profiles of cerebrospinal fluid pathogens in children with acute bacterial meningitis in Yunnan province, China, 2012-2015
Source: PLoS One. 2017 Jun 29;12(6):e0180161. doi: 10.1371/journal.pone.0180161 (PMC5491142; doi:10.1371/journal.pone.0180161)
Supplement: S1 Table — (DOC) [file pone.0180161.s001.doc]

| **Pathogens** | **2012 N (%)** | **2013 N (%)** | **2014 N (%)** | **2015 N (%)** | **Total N (%)** |
| --- | --- | --- | --- | --- | --- |
| Gram-positive organisms |  |  |  |  | 91(50.8) |
| *Streptococcus pneumoniae* | 4(2.2) | 10(5.6) | 8(4.4) | 10(5.6) | 32(17.8) |
| *Staphylococcus epidermidis* | 2(1.1) | 5(2.9) | 6(3.3) | 5(2.9) | 18(10.0) |
| *Group B Streptococcus* | 2(1.1) | 2(1.1) | 4(2.2) | 5(2.9) | 13(7.2) |
| *Staphylococcus haemolyticus* | 2(1.1) | 2(1.1) | 3(1.6) | 1(0.6) | 8(4.4) |
| *Group D Streptococcus* | 1(0.6) | 1(0.6) | 2(1.1) | 2(1.1) | 6(3.4) |
| *Staphylococcus aureus* | 1(0.6) | 1(0.6) | 2(1.1) | 1(0.6) | 5(2.9) |
| *Staphylococcus hominis* | 0(0.0) | 2(1.1) | 1(0.6) | 2(1.1) | 5(2.9) |
| *Listeria monocytogenes* | 0(0.0) | 0(0.0) | 2(1.1) | 2(1.1) | 4(2.2) |
| Gram-negative organisms |  |  |  |  | 88(49.2) |
| *E. coli* | 5(2.9) | 13(7.3) | 15(8.4) | 18 (10.1) | 51(28.5) |
| *Haemophilusinfluenzaetype b* | 2(1.1) | 5(2.9) | 4(2.2) | 6(3.3) | 17(9.5) |
| *S. entericaserovarTyphimurium* | 1(0.6) | 2(1.1) | 2(1.1) | 2(1.1) | 7(3.9) |
| *Klebsiellapneumoniae* | 0(0.0) | 1(0.6) | 1(0.6) | 1(0.6) | 3(1.7) |
| *Pseudomonas aeruginosa* | 1(0.6) | 1(0.6) | 1(0.6) | 0(0.0) | 3(1.7) |
| *Moraxella catarrhalis* | 0(0.0) | 0(0.0) | 2(1.1) | 1(0.6) | 3(1.7) |
| *Acinetobacterbaumannii* | 0(0.0) | 0(0.0) | 1(0.6) | 1(0.6) | 2(1.1) |
| *Acinetobacterlwoffii* | 0(0.0) | 0(0.0) | 1(0.6) | 1(0.6) | 2(1.1) |
| Total | 21(11.7) | 45(25.1) | 55(30.7) | 58(32.5) | 179(100.0) |
