# Supplementary material for: Prevalence and antibiotic resistance profiles of cerebrospinal fluid pathogens in children with acute bacterial meningitis in Yunnan province, China, 2012-2015
Source: PLoS One. 2017 Jun 29;12(6):e0180161. doi: 10.1371/journal.pone.0180161 (PMC5491142; doi:10.1371/journal.pone.0180161)
Supplement: S2 Table — (DOC) [file pone.0180161.s002.doc]

| **Clinical Symptoms** | **≤28 days** | **>28days**  **≤3 months** | **>3months**  **≤1 ages** | **>1ages**  **≤3ages** | **>3ages**  **≤12 ages** | **Number of Patients** | **Occurrence (%)** |
| --- | --- | --- | --- | --- | --- | --- | --- |
| Fever | 40 | 36 | 53 | 27 | 23 | 179 | 100 |
| Vomiting | 32 | 31 | 43 | 14 | 15 | 135 | 75.4 |
| Meninges irritation | 28 | 27 | 39 | 11 | 11 | 116 | 64.8 |
| Seizure | 18 | 17 | 23 | 9 | 8 | 75 | 41.8 |
| Lethargy | 16 | 15 | 21 | 7 | 6 | 65 | 36.3 |
| Headache | 3 | 3 | 6 | 8 | 7 | 27 | 15.1 |
| Stupor | 5 | 3 | 5 | 3 | 2 | 18 | 10.1 |
| Coma | 3 | 2 | 3 | 1 | 1 | 10 | 5.6 |
