# Supplementary material for: Prevalence and antibiotic resistance profiles of cerebrospinal fluid pathogens in children with acute bacterial meningitis in Yunnan province, China, 2012-2015
Source: PLoS One. 2017 Jun 29;12(6):e0180161. doi: 10.1371/journal.pone.0180161 (PMC5491142; doi:10.1371/journal.pone.0180161)
Supplement: S3 Table — (DOC) [file pone.0180161.s003.doc]

| **Isolate** | **≤28 days**  **a/b (%)** | **>28days**  **--3 months**  **a/b (%)** | **>3months**  **≤1 ages**  **a/b (%)** | **>1ages**  **≤3ages**  **a/b (%)** | **>3ages**  **≤12 ages**  **a/b (%)** | **All ages**  **a/b (%)** |
| --- | --- | --- | --- | --- | --- | --- |
| Gram-positive organisms |  |  |  |  |  | 91/179 (50.8) |
| *Streptococcus pneumoniae* | 4/179(2.2) | 4/179 (2.2) | 16/179 (8.8) | 5/179 (1.1) | 3/179 (0.0) | 32/179 (17.8) |
| *Staphylococcus epidermidis* | 0/179(0.0) | 0/179 (0.0) | 8/179 (4.4) | 3/179 (1.7) | 7/179 (3.9) | 18/179 (10.0) |
| Group B *Streptococcus* | 6/179 (3.3) | 7/179 (3.9) | 0/179 (0.0) | 0/179 (0.0) | 0/179 (0.0) | 13/179 (7.2) |
| *Staphylococcus haemolyticus* | 5/179 (2.9) | 0/179 (0.0) | 1/179 (0.6) | 1/179 (0.6) | 1/179 (0.6) | 8/179 (4.4) |
| Group D *Streptococcus* | 0/179 (0.0) | 0/179 (0.0) | 2/179 (1.1) | 2/179 (1.1) | 2/179 (1.1) | 6/179 (3.4) |
| *Staphylococcus aureus* | 0/179 (0.0) | 1/179 (0.6) | 1/179 (0.6) | 1/179 (0.6) | 2/179 (1.1) | 5/179 (2.9) |
| *Staphylococcus hominis* | 0/179 (0.0) | 2/179 (1.1) | 1/179 (0.6) | 1/179 (0.6) | 1/179 (0.6) | 5/179 (2.9) |
| *Listeria monocytogenes* | 0/179 (0.0) | 0/179 (0.0) | 2/179 (1.1) | 2/179 (1.1) | 0/179 (0.0) | 4/179 (2.2) |
| Gram-negative organisms |  |  |  |  |  | 88/179 (49.2) |
| *E. coli* | 20/179 (11.3) | 19/179 (8.8) | 11/179 (4.4) | 0/179 (0.0) | 1/179 (0.6) | 51/179 (28.5) |
| *Haemophilus influenzae* type b | 1/179 (0.6) | 0/179 (0.0) | 8/179 (4.4) | 8/179 (4.4) | 0/179 (0.0) | 17/179 (9.5) |
| *S.entericaserovar Typhimurium* | 1/179 (0.6) | 0/179 (0.0) | 2/179 (1.1) | 2/179 (1.1) | 2/179 (1.1) | 7/179 (3.9) |
| *Klebsiella pneumoniae* | 1/179 (0.6) | 2/179 (1.1) | 0/179 (0.0) | 0/179 (0.0) | 0/179 (0.0) | 3/179 (1.7) |
| *Pseudomonas aeruginosa* | 2/179 (1.1) | 1/179 (0.6) | 0/179 (0.0) | 0/179 (0.0) | 0/179 (0.0) | 3/179 (1.7) |
| *Moraxella catarrhalis* | 0/179 (0.0) | 0/179 (0.0) | 0/179 (0.0) | 0/179 (0.0) | 3/179 (1.7) | 3/179 (1.7) |
| *Acinetobacter baumannii* | 0/179 (0.0) | 0/179 (0.0) | 1/179 (0.6) | 1/179 (0.6) | 0/179 (0.0) | 2/179 (1.1) |
| *Acinetobacter lwoffii* | 0/179 (0.0) | 0/179 (0.0) | 0/179 (0.0) | 1/179 (0.6) | 1/179 (0.6) | 2/179 (1.1) |
| Total | 40/179 (22.4) | 36/179 (20.1) | 53/179 (29.6) | 27/179 (15.1) | 23/179 (12.8) | 179/179 (100.0) |
