# Supplementary material for: Prevalence and antibiotic resistance profiles of cerebrospinal fluid pathogens in children with acute bacterial meningitis in Yunnan province, China, 2012-2015
Source: PLoS One. 2017 Jun 29;12(6):e0180161. doi: 10.1371/journal.pone.0180161 (PMC5491142; doi:10.1371/journal.pone.0180161)
Supplement: S4 Table — (DOC) [file pone.0180161.s004.doc]

| **Antimicrobial** | ***Streptococcus pneumoniae***  *a/b (%)* | ***Staphylococcus epidermidis***  *a/b (%)* | ***GroupB***  ***Streptococcus***  *a/b (%)* | ***Staphylococcus haemolyticus***  *a/b (%)* | ***GroupD Streptococcus***  *a/b (%)* | ***Staphylococcus***  ***Aureus***  *a/b (%)* | ***Staphylococcus***  ***Hominis***  *a/b (%)* | ***Listeria monocytogenes*** *a/b (%)* |
| --- | --- | --- | --- | --- | --- | --- | --- | --- |
| Penicillin G | 20/32(68.8) | 0/18(0.0) | 13/13(100.0) | 0/8(0.0) | 0/6(0.0) | 0/5(0.0) | 1/5 (20.0) | 0/4(0.0) |
| Oxacillin | – | 6/18(33.3) | – | 0/8(0.0) | 0/6 (0.0) | 3/5 (60.0) | 2/5(40.0) | 3/4 (75.0) |
| Gentamicin | 32/32 (100.0) | 14/18(77.8) | – | 4/8(50.0) | 0/6 (0.0) | 5/5 (100.0) | 5/5(100.0) | 3/4 (75.0) |
| Rifampicin | – | 16/18(88.9) | – | 6/8(75.0) | 4/6 (66.7) | 5/5 (100.0) | 5/5 (100.0) | – |
| Ciprofloxacin | – | 10/18(55.6) | 9/13(69.2) | 2/8(25.0) | 4/6 (66.7) | 5/5 (100.0) | 3/5 (60.0) | 4/4 (100.0) |
| Moxifloxacin | – | 8/18(44.4) | – | 2/8(25.0) | – | 5/5 (100.0) | 3/5(60.0) | – |
| Norfloxacin | – | 10/18(55.6) | – | 1/8(12.5) | 4/6 (66.7) | 5/5 (100.0) | 3/5(60.0) | 4/4 (100.0) |
| Ofloxacin | 32/32(100.0) | 8/18(44.4) | 11/13(84.6) | 0/8(0.0) | 3/6 (50.0) | 5/5 (100.0) | 3/5(60.0) | 4/4 (100.0) |
| Levofloxacin | 28/32(87.5) | 10/18(55.6) | 10/13(76.9) | 1/8(12.5) | 3/6 (50.0) | 5/5 (100.0) | 3/5(60.0 | 4/4 (100.0) |
| Co-trimoxazole | 0/32(0.0) | 9/18(50.0) | – | 4/8(50.0) | 4/6 (66.7) | 3/5 (60.0) | 2/5(40.0) | – |
| Clindamycin | 0/32(0.0) | 11/18(61.1) | 3/13(23.1) | 3/8(37.5) | 4/6 (66.7) | 1/5 (20.0) | 2/5(40.0) | – |
| Erythromycin | 0/32(0.0) | 1/18(5.6) | 1/13(7.7) | 0/8(0.0) | 0/6 (0.0) | 1/5 (20.0) | 0/5(0.0) | 2/4 (50.0) |
| Linezolid | 32/32(100.0) | 18/18(100.0) | 13/13(100.0) | 8/8(100.0) | 6/6 (100.0) | 5/5 (100.0) | 5/5(100.0) | 4/4 (100.0) |
| Vancomycin | 32/32(100.0) | 18/18(100.0) | 13/13(100.0) | 8/8(100.0) | 5/6 (83.3) | 5/5 (100.0) | 5/5 (100.0) | 4/4 (100.0) |
| Chloramphenicol | 28/32(87.5) | 15/18(83.3) | 11/13(84.6) | 7/8(87.5) | 4/6 (66.7) | 3/5 (60.0) | 4/5 (80.0) | – |
| Tetracycline | 0/32(0.0) | 6/18(33.3) | 0/13(0.0) | 4/8(50.0) | 1/6 (16.7) | 1/5 (20.0) | 3/5 (60.0) | – |
| Ampicillin | – | 0/18(0.0) | 13/13(100.0) | 0/8(0.0) | 3/6 (50.0) | 0/5 (0.0) | 1/5 (20.0) | 2/4 (50.0) |
| Amoxicillin+clavulanate | – | 5/18(27.7) | – | 2/8(25.0) | – | 2/5 (40.0) | 2/5 (40.0) | – |
| Ceftriaxone | 26/32(81.3) | – | 13/13(100.0) | – | 0/6 (0.0) | – | – | 4/4 (100.0) |
| Meropenem | 24/32(75.0) | – | 13/13(100.0) | – | – | – | – | – |
| Azithromycin | – | 0/18(0.0) | – | 0/8(0.0) | – | 0/5 (0.0) | 0/5 (0.0) | – |
| Clarithromycin | – | 3/18(16.6) | – | 2/8(25.0) | – | 4/5 (80.0) | 3/5 (60.0) | – |
| Total | 32 | 18 | 13 | 8 | 6 | 5 | 5 | 4 |
