# Supplementary material for: Prevalence and antibiotic resistance profiles of cerebrospinal fluid pathogens in children with acute bacterial meningitis in Yunnan province, China, 2012-2015
Source: PLoS One. 2017 Jun 29;12(6):e0180161. doi: 10.1371/journal.pone.0180161 (PMC5491142; doi:10.1371/journal.pone.0180161)
Supplement: S5 Table — (DOC) [file pone.0180161.s005.doc]

| **Antimicrobial** | ***E. coli***  ***a/b (%)*** | ***Haemophilus influenzae type b***  ***a/b (%)*** | ***S. enterica serovar Typhimurium***  ***a/b (%)*** | ***Klebsiella pneumoniae***  ***a/b (%)*** | ***Pseudomonas aeruginosa***  ***a/b (%)*** | ***Moraxella catarrhalis***  ***a/b (%)*** | ***Acinetobacter baumannii***  ***a/b (%)*** | ***Acinetobacter lwoffii***  ***a/b (%)*** |
| --- | --- | --- | --- | --- | --- | --- | --- | --- |
| Ampicillin | 2/51(3.9) | 1/17(5.8) | 0/7(0.0) | 0/3(0.0) | – | 0/3(0.0) | – | – |
| Piperacillin | 8/51(15.7) | – | – | – | 2/3(66.7) | – | 1/2(50.0) | 1/2(50.0) |
| Ampicillin/sulbactam | 15/51(29.4) | – | – | 3/3(100.0) | 1/3(33.3) | 2/3(66.6) | – | – |
| Piperacillin/Tazobactam | 44/51(86.3) | – | – | 3/3(100.0) | 3/3(100.0) | – | 2/2(100.0) | 1/2(50.0) |
| Cefoperazone | 15/51(29.4) | 6/17(35.2) | 3/7 (42.8) | 1/3(33.3) | – | 2/3(66.6) | 0/2(0.0) | 0/2(0.0) |
| Cefuroxime | 22/51(43.1) | 14/17(82.4) | 2/7(28.5) | 1/3(33.3) | – | 1/3(33.3) | 0/2(0.0) | 0/2(0.0) |
| Ceftazidime | 34/51(66.6) | 14/17(82.4) | 3/7 (42.8) | 1/3(33.3) | 2/3(66.7) | 2/3(66.6) | 0/2(0.0) | 0/2(0.0) |
| Ceftriaxone | 22/51(43.1) | 14/17(82.4) | 2/7 (28.5) | 1/3(33.3) | 0/3(0.0) | 1/3(33.3) | 0/2(0.0) | 0/2(0.0) |
| Cefepime | 34/51(66.6) | 16/17(94.1) | 4/7 (57.1) | 2/3(66.6) | 2/3(66.7) | 3/3(100.0) | 0/2(0.0) | 0/2(0.0) |
| Cefoxitin | 44/51(78.4) | – | – | 1/3(33.3) | – | – | – | – |
| Aztreonam | 25/51(49.0) | – | – | – | 2/3(66.7) | – | – | – |
| Imipenem | 51/51(100.0) | 17/17(100.0) | 7/7(100.0) | 3/3(100.0) | 3/3(100.0) | 3/3(100.0) | 2/2(100.0) | 2/2(100.0) |
| Meropenem | 51/51(100.0) | 17/17(100.0) | 7/7(100.0) | 3/3(100.0) | 3/3(100.0) | 3/3(100.0) | 2/2(100.0) | 2/2(100.0) |
| Amikacin | 51/51(100.0) | 4/17(23.5) | 7/7(100.0) | 1/3(33.3) | 3/3(100.0) | 3/3(100.0) | 1/2(50.0) | 1/2(50.0) |
| Gentamicin | 30/51(58.8) | 15/17(88.2) | 6/7(85.7) | 1/3(33.3) | 3/3(100.0) | – | 1/2(50.0) | 1/2(50.0) |
| Tobramycin | 38/51(74.5) | – | 6/7(85.7) | – | 3/3(100.0) | – | 0/2(0.0) | 0/2(0.0) |
| Ciprofloxacin | 32/51(62.7) | 16/17(94.1) | – | 2/3(66.6) | 3/3(100.0) | 3/3(100.0) | 1/2(50.0) | 1/2(50.0) |
| Levofloxacin | 30/51(58.8) | – | 5/7(71.4) | 2/3(66.6) | 3/3(100.0) | 3/3(100.0) | 1/2(50.0) | 1/2(50.0) |
| Norfloxacin | 29/51(56.8) | – | 6/7(85.7) | – | – | 3/3(100.0) | – | – |
| Co-trimoxazole | 16/51(31.4) | 4/17(23.5) | 1/7(14.2) | 1/3(33.3) | – | – | 0/2(0.0) | 0/2(0.0) |
| Chloramphenicol | 45/51(88.2) | 17/17(100.0) | 1/7(14.2) | 3/3(100.0) | 1/3(33.3) | – | 1/2(50.0) | 0/2(0.0) |
| Minocycline | 38/51(74.5) | – | – | – | – | 3/3(100.0) | – | – |
| Total | 51 | 17 | 7 | 3 | 3 | 3 | 2 | 2 |
